# Determination of the Minimum Discard Volume in the Extraction of Blood Samples Through Arterial Catheters in Critical Patients

**DOI:** 10.1111/nicc.70493

**Published:** 2026-04-15

**Authors:** Patricia Morales Fernández, Francisco Javier Castro Olmo, Sara González‐Martín, Miguel Serrano Hernández, Manuel Quintana Díaz, Víctor Aceña Gil, Estefani Martínez‐Chávez, Belén Fernández‐Puntero

**Affiliations:** ^1^ Burn Unit Hospital Universitario de La Paz Madrid Spain; ^2^ Faculty of Health Sciences Universidad Rey Juan Carlos Alcorcón Spain; ^3^ Department of Intensive Care Medicine Hospital Universitario de La Paz Madrid Spain; ^4^ Data Science Laboratory Universidad Rey Juan Carlos Móstoles Spain; ^5^ Clinical Analysis Service Hospital Universitario de La Paz Madrid Spain

## Abstract

**Background:**

The critically ill patient requires multiple extractions through catheters, implying a substantial blood loss. The discard method continues to be a commonly used method in Intensive Care Units. However, there is no homogeneity in practice.

**Aim:**

Determine the minimum blood discard volume to obtain samples through radial arterial catheter.

**Study Design:**

Quasi‐experimental, prospective, cross‐sectional study employing the STOBE checklist. Four consecutive arterial blood gases were performed considering the flush volume (sample A with 1 dead space discarding 1 mL, sample B with 2 discard space [2 mL], sample C with 3 discard space [3 mL] and control with 4 discard space [4 mL]). The study was approved by the Ethics and Pharmacological Research Committee of the Research Institute of La paz Hospital (Law No. 2023.564). It was conducted in the Burn Intensive Care Unit and the Resuscitation and Critical Care Unit of La Paz Hospital from September 2023 to June 2024.

**Results:**

We found values outside the Clinical Acceptance Interval (CAI) in sample A versus control in pCO_2_ (47.42%), HCO_3_^−^ (60.71%), Haemoglobin (67.85%), Na^2+^ (7.14%), K^+^ (71.42%) and glycaemia (21.42%). In the case of lactate, no values outside the Clinical Acceptance Interval were found. When comparing sample B versus control, we found significant differences in HCO_3_^−^ (*p* = 0.027) and glycaemia (*p* = 0.001). However, none of the parameters studied presented values outside the Clinical Acceptance Interval. When the results were related to the Reference Value of Change (RVC), the pCO_2_ and Haemoglobin values presented 3.57% of values outside the Clinical Acceptance Interval, respectively, although they did not present statistically significant differences (*p* > 0.05). When comparing sample C versus control, no significant differences were found in any parameter, nor values outside the Clinical Acceptance Interval.

**Conclusions:**

The volume of discard required to obtain valid values is twice the volume used to purge the arterial catheterization system (2 mL) for obtaining samples for clinical use.

**Relevance to Clinical Practice:**

The calculation of the minimum amount of discard in the collection of blood samples through the arterial catheter is an intervention at no extra cost that will reduce iatrogenic anaemia, reducing the need for transfusions, the complications associated with this therapy and the reduction of costs in critically ill patients.

## Impact Statement

- What is known about the topic
  ○ Critical patients require constant blood determinations which imply consumption and loss of blood, both for the volume extracted for the analysis itself and for the amount of waste blood prior to obtaining the sample.
  ○ The method most commonly used in ICUs is the discard method. The literature includes wide variability in the recommendations on the volume of discarded blood.
- What this paper adds
  ○ Determine the volume discard of blood samples in relation to the catheter used.
  ○ Decrease discard volume after blood extraction through an arterial catheter.
  ○ Use the Clinical Acceptance Interval (CAI) and the Reference Value of Change (RVC) to assess the clinical utility of the results (allowing the results to be safely extrapolated to the clinical setting).

## 1 Introduction

Critical patients require constant monitoring and multiple invasive therapies for their care and survival. This includes numerous blood determinations [1, 2], which entail both consumption and loss of blood, both for the volume extracted for the analysis itself and for the amount of waste blood prior to obtaining the sample [3, 4].

Blood sampling may be performed either by direct puncture (without prior discard) or through an indwelling catheter, thereby reducing the number of punctures required.

In critically ill patients, blood draws are usually performed from a central venous catheter or from an arterial catheter. Tarpey and Lawler noted that patients with arterial catheters are phlebotomized twice as often as those without such catheterization and that blood loss is three times greater in catheterized patients than in patients who do not have intra‐arterial access [5].

Clinical practice guidelines identify arterial blood as the reference standard for the assessment of gas exchange, glycaemic control and a wide range of additional parameters [6, 7, 8]. To ensure the validity of these measurements, it is essential to confirm that the collected sample has not been altered by external factors, such as the infusion of medications through the same catheter. Consequently, the discard method is commonly employed [9] which involves removing and discarding a predetermined volume of blood prior to sample collection in order to prevent contamination or dilution. As a result, blood sampling entails not only the extraction of the volume required for laboratory analysis but also an additional discard volume that is not reinfused and is ultimately wasted.

The average increase in the volume of blood drawn for analysis in 5 mL of blood has been identified by Bodley et al. as the main predictor of anaemia acquired in intensive care units and the need for red blood cell transfusion, which is associated with an increase of more than 1 point in the SOFA score on Day 1 [10]. In‐hospital acquired anaemia (HAA) represents one of the main complications in critically ill patients [5, 11, 12], since it increases both the length of stay in the intensive care units and subsequent hospitalization as well as mortality. This problem is associated with the risks linked to blood transfusions in anaemic patients who require this treatment, which highlights the importance of *patient blood management* programmes [11, 12].

Among the interventions aimed at reducing this blood loss associated with the extraction of blood samples, the use of non‐invasive tests, such as capnography or pulse oximetry; *point‐of‐care testing* [13], which requires a smaller sample volume; tubes of smaller capacity [6]; and blood preservation devices [1, 2, 3, 4, 13, 14, 15, 16, 17]. The implementation of these strategies, either individually or through integrated patient blood management protocols [18], varies across healthcare systems [19].

In clinical practice, the use of closed loop blood sampling *systems* is not widespread, although they are identified in the literature as devices that are recommended for routine care [20]. The method most commonly used in intensive care units is the discard method [9]. However, there is no homogeneity regarding the volume of blood to be discarded, which generates variability between different nursing units and even between professionals.

It is estimated that between 28.5% and 33.1% of the total volume of blood drawn for analysis corresponds to discarded blood [9] with an average of 31.61 mL discarded during the first 24 h of the admission of a critical patient [21]. The mean volume of blood discarded per extraction is 10.3 mL, which represents an average discard volume of 41.1 mL per day [22].

This fact reflects a point of improvement related to patient safety, since the reduction in the volume of discarded blood could contribute to reducing the incidence of hospital acquired anaemia and, therefore, the need for the transfusion of blood products, which has a higher risk of morbidity and mortality [23].

## 2 Background/Justification for Study

The literature includes wide variability in the recommendations on the volume of discarded blood. Some sources suggest discarding a volume equivalent to the dead space of the catheter and the system [22], whereas others recommend a discard volume of three times the dead space [24], between 5 and 10 mL [25, 26], six times the dead space [27] or twice the volume of the catheter's dead space [28]. In addition, in cases of infusion with sodium heparin, discarding a larger volume is suggested, although there are also discrepancies in the recommendations in this regard [29]. The evidence is based on extraction in venous catheters [24, 25, 26, 27, 28, 29], without taking into account the peculiarities of arterial catheterization. Therefore, it is crucial to establish a standardized arterial discard volume that minimizes blood loss while ensuring the accurate measurement of all parameters required for the assessment, management and treatment of critically ill patients.

With respect to the discarding technique, some studies suggest prior washing for access with physiological saline [25] or other unspecified solutions. As a result, there is great variability in the volume of blood discarded for obtaining samples, exceeding the minimum recommended volume on many occasions.

Given the importance of in‐hospital iatrogenic anaemia and its impact on the evolution of critical patients, minimizing, as much as possible, all interventions that favour its appearance is highly important. One of these interventions is the optimization of the discard volume in each blood extraction. Reducing this volume could help prevent iatrogenic anaemia, promote blood savings and reduce the need for transfusions, with a consequent positive impact on reducing complications.

## 3 Aim

The general aim of this study was to determine the minimum volume of discarded blood necessary to obtain blood samples through a radial arterial catheter, establishing a multiple of the dead space that is valid for the measurement of the parameters of commonly used arterial blood gases and electrolytes.

## 4 Design and Methods

This was a quasi‐experimental, prospective and cross‐sectional study. The study was conducted in a real clinical setting involving intensive care unit patients. This study employed The Strengthening the Reporting of Observational Studies in Epidemiology checklist.

### 4.1 Setting and Sample

The Burn Intensive Care Unit (ICU) and the Resuscitation and Critical Care Unit of the La Paz Hospital, from September 2023 to June 2024.

### 4.2 Participants

Patients admitted to both units with a temporary arterial catheter housed in the radial artery with haemoglobin values > 7 g/dL were included. The exclusion criteria were patients under 18 years of age who received continuous heparin infusion.

This study deliberately focussed on radial arterial catheterization for hemodynamic monitoring. This decision was based on the considerable heterogeneity of arterial catheter systems currently available, which extends beyond differences in manufacturers to include variations in catheter function (e.g., haemodynamic monitoring vs. other clinical purposes) and anatomical insertion sites (radial, femoral or brachial arteries). These factors inherently influence key technical characteristics such as catheter length, gauge and overall system dead space, all of which are directly relevant to the determination of appropriate discard volumes. By restricting the study to radial arterial catheters in adult patients, we aimed to enhance internal validity through the use of a relatively homogeneous and widely utilized access site that is routinely managed by nursing professionals in both insertion and maintenance. This approach provides a controlled framework for evaluating discard volume while establishing a foundation for future research involving other arterial access sites and catheter types, ultimately contributing to the development of standardized, safe and generalizable protocols for arterial blood sampling across diverse clinical settings.

### 4.3 Sample Size Calculation/Sample

The sample size was calculated for a significance level of *α* = 0.05 and a power (1 − *β*) = 0.90. The necessary calculations were carried out using the statistical software GPower^15^ version 3.1.9.6, applying the non‐centrality parameter formula for repeated measures: $\lambda =f^{2}N\;\left(\frac{m}{1-\rho }\right).$ Specifically, the calculation was set to detect a small effect size (f=0.15) considering m=4 measurements, an alpha of 0.05, a power of 0.90, and a conservative correlation among repeated measures of ρ=0.81, establishing a required minimum of N=31 patients.

A study was performed among patients admitted to the intensive care unit and reanimation units. The total number of participating patients was 31, although three of them were excluded for not having all the samples necessary for their participation, resulting in a final sample of 28 subjects.

### 4.4 Variables

The following blood parameters commonly analysed in arterial blood gas were used: pH, partial pressure of oxygen (pO_2_), partial pressure of carbon dioxide (pCO_2_), bicarbonate (HCO_3_^−^), haemoglobin (Hb), sodium (Na^2+^), potassium (K^+^), glycaemia (Glu) and lactate (Lac).

Although severity scores such as APACHE II were not collected, the within‐subject design ensures that all samples were obtained from the same patient during a single extraction procedure, minimizing variability related to clinical status.

### 4.5 Data Collection Tools and Methods

The samples were obtained by five nurses previously trained in the extraction and analysis of arterial blood gases in the *point‐of‐care testing* equipment of the units themselves in accordance with the hospital's internal protocol.

Four consecutive arterial blood gas measurements were performed through the three‐way port integrated in the artery transducer system (*Meritrans DTX Plus*, Merck Medical Systems, USA).

To define the intervention volumes, the radial arterial catheterization sets (with dimensions of 20G and 8 cm and a priming volume of 0.17 mL) and the dead space were considered, that is, the volume from the tip of the catheter to the extraction port, including a bioconnector (*MicroClave Clear Connector*, ICU Medical, USA), was approximately 1 mL (0.17 mL catheter +0.8 mL part corresponding to the system).

The discard volumes, once approximated to integer values, for the study were as follows:

- **Sample A:** one time the discard volume (1 mL)
- **Sample B:** two times the discard volume (2 mL)
- **Sample C:** three times the discard volume (3 mL)
- **Control sample:** 4 mL (reference value, based on the average of the usual discard volume: 4.37 ± 2.93 mL)

The procedure was carried out under aseptic conditions, following the recommendations of the Zero Bacteremia Project. The patency of the catheters was maintained by a physiological saline solution pressurized at 300 mmHg, with a constant infusion rate of 3 mL/h.

The sampling process consisted of the following steps: Sampling order was fixed (A, B, C, Control) and flushing between samples was not performed to simulate standard clinical practice

1. One millilitre of blood was discarded via a standard 2 mL syringe.
2. Four samples (A, B, C and the control), each with a volume of 1 mL, were obtained consecutively. The total volume of blood drawn per patient was 5 mL.

Once the three intervention samples and the control sample had been obtained, the subject's participation was terminated.

The samples were immediately analysed via a gasometer (*ABL90 Flex*, Radiometer Medical, Denmark) located in the units themselves.

### 4.6 Data Analysis

All the statistical analyses were performed with the R program (version 4.4.0). The level of significance used was defined as α = 0.05 for a 95% confidence interval (CI).

To evaluate the normality of the data, the Shapiro–Wilk test was applied. To analyse between‐group differences, repeated‐measures ANOVA was used. In cases where the sphericity assumption was not met, the Greenhouse–Geisser correction was applied. When significant differences were detected, a *post hoc* comparison was used with the Bonferroni correction.

To evaluate the clinical significance of the observed differences, the reference value of change (RVC), which defined the Clinical Acceptance Interval (CAI) between the intervention samples (A, B, and C) and the control sample, was used.

The calculation of the reference value of change was carried out through the calculator of the European Biological Variation Database [30]. This statistical tool integrates the coefficient of analytical variation (CAV) and the individual biological variation (CIV), which allows us to determine if the differences between the samples can be attributed to analytical and biological variations or if they reflect a clinically relevant change in the patient's condition.

The individual biological variation values were obtained from the European biological variation database [30], whereas the coefficient of analytical variation values were obtained from the internal quality control of the laboratory for each of the parameters analysed.

When the difference between an intervention sample and the control sample exceeds the reference value of change, the observed change cannot be explained by analytical or biological variability, which implies that the samples are not equivalent.

Therefore, we consider that the sample may be considered equal to the control if the difference is not statistically significant (*p* > 0.05) and if all values are within the Clinical Acceptance Interval.

The analysis of results through the reference value of change and Clinical Acceptance Interval is essential to assess whether samples that do not show significant differences from the control sample represent clinically relevant differences, thus ensuring that the samples obtained are reliable for clinical decision‐making.

## 5 Ethical and Institutional Approvals

The study was approved by the Ethics and Drug Research Committee of the Hospital La Paz Research Institute (Act n° 16/2023) on September 8, 2023. All the participants provided informed consent after receiving detailed information about the study and before the samples were obtained. The data were treated confidentially according to Organic Law 3/2018, of December 5, on the protection of personal data and guarantee of digital rights both in the collection and in the analysis and dissemination of the results.

## 6 Results

The final sample consisted of 28 patients, of which 64% were men and 36% were women, with ages ranging between 23 and 84 years.

**FIGURE 1:**
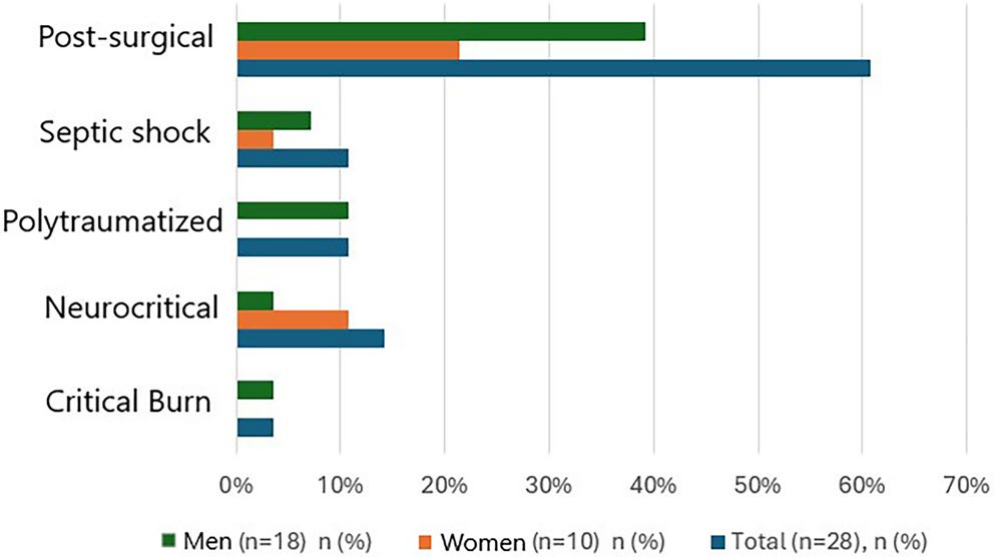
Sociodemographic characteristics of the sample.

The most frequent reason for admission was postsurgical intensive care (60.71%), followed by neurocritical patients (14.28%), polytraumatized patients and those with septic shock (10.71%), and to a lesser extent, critical burn patients (3.57%) (Figure 1).

**FIGURE 2:**
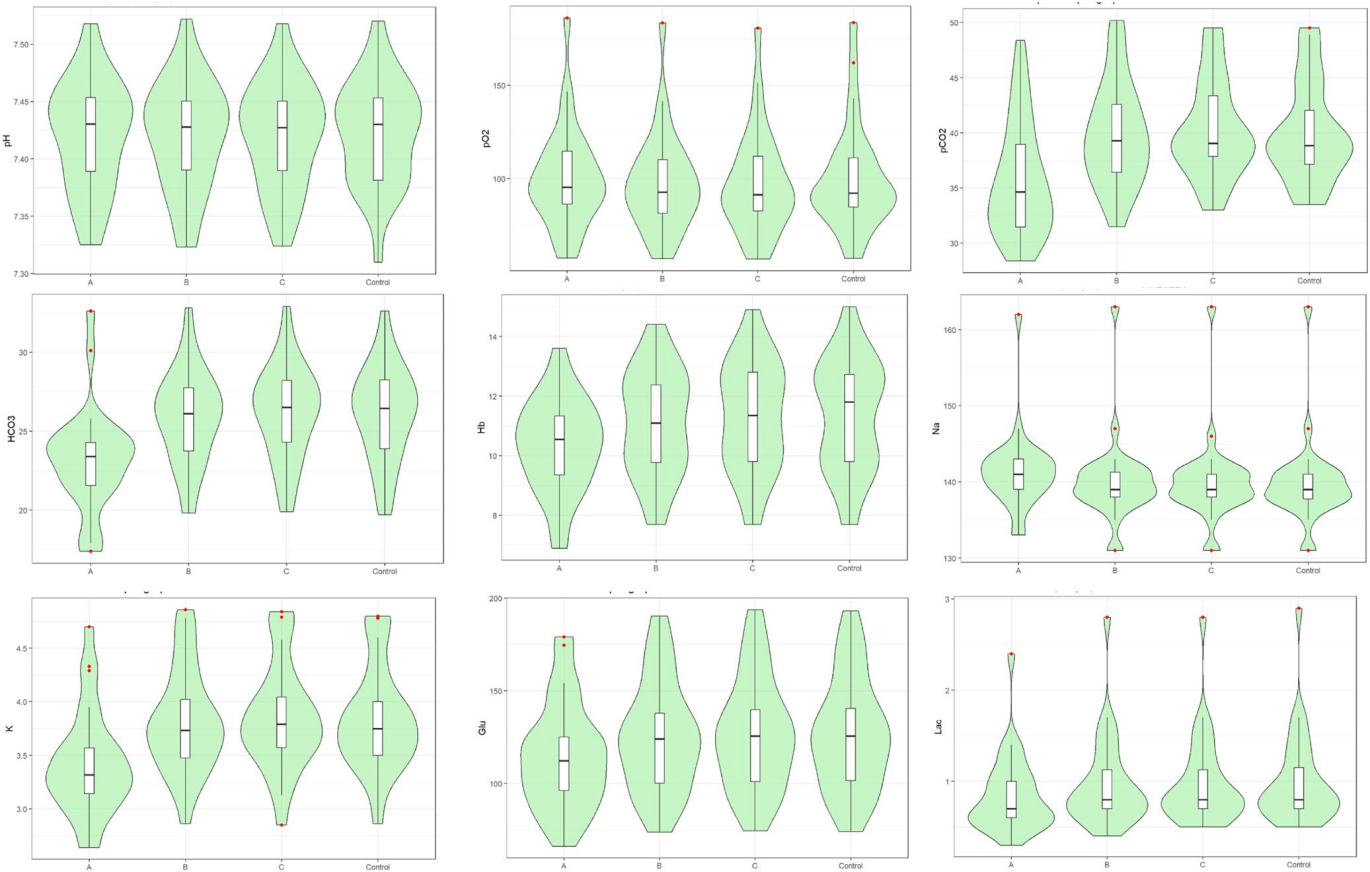
Distribution of blood parameters in the intervention samples (A, B, C) and the control sample. Glu, glycaemia; Hb, haemoglobin; HCO^3−^, bicarbonate ion; K^+^, Potassium; Na^2+^, Sodium; Lac, lactate; pO_2_, partial pressure of oxygen in blood; pCO_2_, partial pressure of carbon dioxide in blood.

The average results of each of the parameters analysed are presented in Figure 2.

Normality analysis revealed that all the variables analysed in the blood samples maintained a normal distribution.

Statistically significant differences between the groups were observed for all the variables, except for pH (Table 1).

**TABLE 1:** Comparison between the intervention samples (A, B, C) and the control sample.

Variable	ANOVA test	Sample A		Sample B		Sample C		Control
	*p*	Median ± SD	*p*	Median ± SD	*p*	Median ± SD	*p*	Median ± SD
pH	0.442*	7.42 ± 0.04	NA	7.42 ± 0.04	NA	7.42 ± 0.04	NA	7.42 ± 0.04
pO_2_	0.016*	100.32 ± 26.97	1	97.70 ± 26.15	0.389	97.67 ± 27.58	0.247	99.94 ± 28.26
pCO_2_	< 0.001*	35.73 ± 5.16	< 0.001*	39.85 ± 4.70	1	40.27 ± 4.41	1	39.99 ± 4.47
HCO_3_^−^	< 0.001*	23.19 ± 3.23	< 0.001*	25.87 ± 2.97	0.027*	26.16 ± 2.99	1	26.13 ± 3.01
Hb	< 0.001*	10.35 ± 1.57	< 0.001*	11.10 ± 1.77	0.064	11.30 ± 1.84	1	11.33 ± 1.88
Na^2+^	< 0.001*	141.28 ± 5.17	< 0.001*	140 ± 5.58	0.1	139.89 ± 5.54	0.618	139.75 ± 5.59
K^+^	< 0.001*	3.42 ± 0.48	1^e‐04^ *	3.81 ± 0.48	1	3.84 ± 0.49	1	3.83 ± 0,49
Glu	< 0.001*	114.54 ± 28.66	< 0.001*	124.97 ± 29.87	0.001*	126.35 ± 30.29	1	126.41 ± 30.49
Lac	< 0.001*	0.85 ± 0.42	< 0.001*	0.97 ± 0.49	0.052	0.98 ± 0.48	0.134	1.00 ± 0.50

Abbreviations: Glu, glycaemia; Hb, Haemoglobin; HCO_3_^−^, bicarbonate ion; K^+^, potassium; Lac, lactate; Na^2+^, sodium; NA, not applicable; pCO_2_, partial pressure of carbon dioxide in blood; pO_2_, partial pressure of oxygen in blood.

* Results with statistically significant differences (*p* < 0.05).

The clinical acceptance interval was calculated with respect to the results of the control sample using the reference value of change (Table 2).

**TABLE 2:** Table with the values outside the CAI by parameter and sample.

Variable	Sample A, *n* (%)	Sample B, *n* (%)	Sample C, *n* (%)
pH	0 (0%)	0 (0%)	0 (0%)
pO_2_	0 (0%)	0 (0%)	0 (0%)
pCO_2_	13 (47, 42%)	1 (3, 57%)	0 (0%)
HCO_3_^−^	17 (60, 71%)	0 (0%)	0 (0%)
Hb	19 (67, 85%)	1 (3, 57%)	0 (0%)
Na^2+^	2 (7, 14%)	0 (0%)	0 (0%)
K^+^	19 (71, 42%)	0 (0%)	0 (0%)
Glu	6 (21, 42%)	0 (0%)	0 (0%)
Lac	0 (0%)	0 (0%)	0 (0%)

Abbreviations: CAI, clinical acceptance interval; Glu, glycaemia; Hb, haemoglobin; HCO_3_^−^, bicarbonate ion; K^+^, potassium; Lac, lactate; Na^2+^, Sodium; pO_2_, partial pressure of oxygen in blood; pCO_2_, partial pressure of carbon dioxide in blood.

In sample A (1 time the discard volume) versus the control, we found a proportion of values outside the Clinical Acceptance Interval for pCO_2_, HCO_3_^−^, haemoglobin, Na^2+^, K^+^ and glycaemia (*p* < 0.001). In the case of pH, pO_2_ and lactate, no values were found outside the range of clinical acceptance. Significant differences were found for all the parameters, except for pH, which did not significantly differ between Group A, B or C and the control.

In the comparison of sample B (twice the discard volume) with the control, significant differences were found in HCO_3_^−^ (*p* = 0.027) and glycaemia (*p* = 0.001). However, none of these *p* values had values outside the Clinical Acceptance Interval. When the results were evaluated with the reference value of change, in the case of sample B (double discard volume) versus the control, the pCO_2_ value and the haemoglobin value presented values outside the Clinical Acceptance Interval, although the differences were not significant (*p* = 1,000 for pCO_2_ and *p* = 0.064 for haemoglobin).

In the comparison between sample C (three times discard volume) and the control sample, no significant differences were found in any of the parameters (*p* > 0.05). Additionally, the values were not outside the Clinical Acceptance Interval for any of the parameters.

These results demonstrate that despite finding statistically significant differences in sample B, these differences do not represent a clinically relevant difference, and sample B can be used without risk.

## 7 Discussion

This study not only confirms the variability in discard volumes described in the literature but also paves the way for a more pathophysiological and context‐specific interpretation of these values. While previous studies, such as those by Arias et al. and O'Hare and Chilvers, place waste volumes in ranges close to 2–3 mL, these data should be interpreted with caution, given that in many cases key variables such as dead space, catheter gauge or catheter length are not specified. This methodological limitation introduces a significant bias, especially when extrapolating results obtained with venous catheters to arterial systems, where flow dynamics, pressure and priming volume differ substantially.

In this context, our findings provide a key element for the synthesis of knowledge: the need to individualize the waste volume based on the physical characteristics of the device. Thus, when considering a 20‐G arterial catheter, 8 cm in length and a priming volume of 0.17 mL, the 2 mL discard volume is not only sufficient from an analytical standpoint but also implies a significant reduction in the volume withdrawn compared to more conservative practices. This approach based on the actual system volume allows for a reinterpretation of published data, suggesting that part of the variability observed in the literature could be explained more by differences in devices than by clinical or analytical criteria.

Comparison with studies on central venous catheters reinforces this hypothesis. The described heterogeneity in discard volumes (ranging from 1.5 to 5 mL) does not appear to follow a uniform logic but rather reflects a lack of standardization regarding the characteristics of the catheter and the infusion system. Therefore, rather than considering these values as direct references, they should be interpreted as indirect evidence of the need for protocols tailored to the specific technical context.

The results obtained are comparable to those of Arias et al., who determined a waste volume of 3 mL for blood gas analysis and biochemistry. O'Hare and Chilves estimated a waste value in arterial catheters, with mean values of 3.2 mL (range 1–7.5 mL) in adult intensive care units, 2.3 mL (range 1–5 mL) in mixed intensive care units and 1.9 mL in paediatric intensive care units [24]. However, comparisons of these results are difficult because the dead space, calibre and length of the evaluated catheters are unknown, as these studies were conducted on venous catheters.

In their survey of 296 nurses from 12 national intensive care units, Pérez‐Juan and Maqueda‐Palau reported a discard volume of 4.37 ± 2.93 mL. However, these data are not relevant to the catheters used [26].

For the comparison of these results, we consider it important to relate the discard volume and the purging volume of the lumen used for extraction, taking into account the size of the arterial catheter used. In our case, the waste volume was 2 mL, considering that the catheters used had a priming volume of 0.17 mL with a length of 8 cm and 20 G with maintenance with physiological saline. This represents a reduction of 2.83 mL for each gasometric extraction.

In studies in which the discard volumes in central venous catheters (CVCs) were evaluated, and disparate volumes, with the use of smaller waste (1.5 and 2 mL) [27, 28], equal smaller waste (3 mL) [29] and higher waste (5 mL) [31], were reported. These results are in line with the evidence obtained, always considering that the same devices are not being used.

From an analytical standpoint, our results also allow us to establish a clear relationship between flush volume and sample reliability. Sample A (1 mL) showed a systematic deviation from the control, with a clinically unacceptable proportion of values outside the acceptance range (more than 60% of the data), especially for critical parameters such as potassium, haemoglobin and bicarbonate. This finding not only confirms the inadequacy of minimum discard volumes but also suggests the persistence of significant contamination by maintenance solution or residual blood in the system.

In contrast, sample B (2 mL) represents a significant transition point. Although statistically significant differences were detected in some parameters (HCO_3_^−^ and glycaemia), these did not reach clinical significance, highlighting the importance of distinguishing between statistical significance and clinical applicability in the intensive care setting. This aspect is particularly relevant in decision‐making, where small analytical variations may not justify an increase in the volume of blood discarded.

In the case of sample C (with a waste volume of 3 mL), the differences relative to the control sample were no significant (*p* > 0.05). This, together with the fact that the values were not outside the Clinical Acceptance Interval, makes it the optimal sample of choice to reduce the discard volume.

Therefore, determining the waste volume based on the catheter's characteristics results in a reduction of waste volume by more than half when comparing our results with those of other studies, such as the one conducted by Maqueda‐Palau [21] in Spanish ICUs, which identified an average waste volume of 4.37 ± 2.93 mL compared to 2 mL, establishing a specific and safe volume based on the catheter used. With this simple standardization of the procedure, we can reduce the current waste volume by 40% through a cost‐free intervention [21].

## 8 Limitations

One of the main strengths of this study lies in its within‐subject design: all samples were collected from the same patient during a single extraction session, minimizing inter‐subject variability. Samples were promptly analysed following established *point‐of‐care testing* protocols by trained personnel, thus ensuring consistency.

The main limitation is that the study was conducted at a single institution using a single catheter type, which may limit the generalizability of the findings. These results should be evaluated in relation to the arterial catheterization system used. Although calculating the discard volume in arterial catheters is an important step, the relationship with the diameter, size of the catheter, and the system used must be taken into account. Future research should explore various catheter models, diameters, and anatomical sites to validate and extend these results.

Blood discarded during arterial sampling represents a substantial and often underestimated source of iatrogenic blood loss in critically ill patients. This variability in clinical practice contributes significantly to hospital‐acquired anaemia, which has been associated with increased morbidity, mortality and healthcare resource utilization. Despite growing emphasis on patient blood management, there remains a lack of standardized recommendations specifically addressing arterial catheter discard volumes.

Our findings support the hypothesis that discard volume can be safely reduced without compromising clinical reliability when it is proportional to the system dead space. This has important implications for patient blood management, as even small reductions per extraction may result in significant cumulative blood savings.

The exclusive use of saline should be considered when interpreting these findings, as heparinized systems may require different discard volumes due to potential analytical interference.

## 9 Implications for Practice

Therefore, future lines of research should evaluate different types of catheters (tunnelled, central venous catheters, catheter with a subcutaneous reservoir) in different locations, especially the radial and femoral arteries, as well as the inclusion of other analytical parameters.

## 10 Conclusions

This study concluded that a discard volume of 2 mL—equivalent to two times the dead space—is sufficient to obtain reliable blood gas and electrolyte values from radial arterial catheters. These findings apply specifically to the catheter configuration evaluated in this study.

The expansion of these studies with conditions that assess other devices and locations would allow the establishment of the minimum volumes of sample discarded in the extractions, contributing to blood management protocols programs to reduce blood loss in adult patients by up to 40% at no additional cost.

## Funding

The authors have nothing to report.

## Ethics Statement

The study was approved by the Ethics and Drug Research Committee of the Hospital Universitario La Paz Research Institute (Act no. 16/2023). All the participants provided informed consent after receiving detailed information about the study and before the samples were obtained. The data were treated confidentially according to Organic Law 3/2018, of December 5, on the protection of personal data and guarantee of digital rights both in the collection and in the analysis and dissemination of the results.

## Consent

All patients were informed and signed the informed consent form before participating in the study.

## Conflicts of Interest

The authors declare no conflicts of interest.

## Data Availability

The data that support the findings of this study are available on request from the corresponding author. The data are not publicly available due to privacy or ethical restrictions.
